# 450K Epigenome-Wide Scan Identifies Differential DNA Methylation in Newborns Related to Maternal Smoking during Pregnancy

**DOI:** 10.1289/ehp.1205412

**Published:** 2012-07-31

**Authors:** Bonnie R. Joubert, Siri E. Håberg, Roy M. Nilsen, Xuting Wang, Stein E. Vollset, Susan K. Murphy, Zhiqing Huang, Cathrine Hoyo, Øivind Midttun, Lea A. Cupul-Uicab, Per M. Ueland, Michael C. Wu, Wenche Nystad, Douglas A. Bell, Shyamal D. Peddada, Stephanie J. London

**Affiliations:** 1 Division of Intramural Research, National Institute of Environmental Health Sciences, National Institutes of Health, Department of Health and Human Services, Research Triangle Park, North Carolina, USA; 2 Norwegian Institute of Public Health, Oslo, Norway; 3 Haukeland University Hospital, Bergen, Norway; 4 University of Bergen, Bergen, Norway; 5 Duke University School of Medicine, Durham, North Carolina, USA; 6 Bevital A/S, Laboratoriebygget, Bergen, Norway; 7 Department of Biostatistics, Gillings School of Global Public Health, University of North Carolina, Chapel Hill, North Carolina, USA

**Keywords:** epigenetics, epigenome-wide, *in utero*, maternal smoking, methylation

## Abstract

Background: Epigenetic modifications, such as DNA methylation, due to *in utero* exposures may play a critical role in early programming for childhood and adult illness. Maternal smoking is a major risk factor for multiple adverse health outcomes in children, but the underlying mechanisms are unclear.

Objective: We investigated epigenome-wide methylation in cord blood of newborns in relation to maternal smoking during pregnancy.

Methods: We examined maternal plasma cotinine (an objective biomarker of smoking) measured during pregnancy in relation to DNA methylation at 473,844 CpG sites (CpGs) in 1,062 newborn cord blood samples from the Norwegian Mother and Child Cohort Study (MoBa) using the Infinium HumanMethylation450 BeadChip (450K).

Results: We found differential DNA methylation at epigenome-wide statistical significance (*p*-value < 1.06 × 10^–7^) for 26 CpGs mapped to 10 genes. We replicated findings for CpGs in *AHRR*, *CYP1A1*, and *GFI1* at strict Bonferroni-corrected statistical significance in a U.S. birth cohort. *AHRR* and *CYP1A1* play a key role in the aryl hydrocarbon receptor signaling pathway, which mediates the detoxification of the components of tobacco smoke. *GFI1* is involved in diverse developmental processes but has not previously been implicated in responses to tobacco smoke.

Conclusions: We identified a set of genes with methylation changes present at birth in children whose mothers smoked during pregnancy. This is the first study of differential methylation across the genome in relation to maternal smoking during pregnancy using the 450K platform. Our findings implicate epigenetic mechanisms in the pathogenesis of the adverse health outcomes associated with this important *in utero* exposure.

Maternal smoking during pregnancy is a major risk factor for adverse health outcomes in children including low birth weight, some childhood cancers, reduced lung function, and early respiratory illnesses (Office of the Surgeon General 2006). Recent evidence suggests that maternal smoking during pregnancy leads to obesity and elevated blood pressure in children (Brion et al. 2008; Cupul-Uicab et al. 2012). The underlying mechanisms for the diverse effects of maternal smoking during pregnancy on offspring may involve epigenetic modifications such as DNA methylation.

DNA cytosine methylation plays a key role in modulating the transcriptional potential of the genome and may influence the development of complex human diseases (Feinberg 2010). Changes to DNA methylation can occur throughout life, but much of the epigenome is established during embryogenesis and early development of the fetus (Reik 2007).

Data from animal models demonstrate that maternal exposures, such as dietary methyl donors (Hollingsworth et al. 2008) and bisphenol A (Dolinoy et al. 2007), can affect offspring phenotypes via DNA methylation. A few human studies have examined epigenetic alterations in relation to maternal smoking during pregnancy and reported it to be associated with global methylation of leukocyte DNA using a [^3^H]-methyl acceptance assay (Terry et al. 2008), global [LINE-1 (long interspersed nuclear element-1) and AluYb8 (short interspersed element)] methylation in human placenta (Wilhelm-Benartzi et al. 2012), or differential methylation at cytosine–guanine dinucleotide (CpG) sites [CpG-specific methylation using the Illumina Infinium HumanMethylation27 Beadchip (27K) (Illumina Inc., San Diego, CA, USA) measuring approximately 27,000 CpGs] in human placenta (Suter et al. 2011). Maternal tobacco use during pregnancy has also been associated with global (LINE-1 and AluYb8) and CpG-specific methylation (Illumina GoldenGate Cancer methylation panel I measuring 1,505 CpGs) in buccal cells from children (Breton et al. 2009). To date, the effect of maternal smoking on differential DNA methylation has not been evaluated with more comprehensive epigenomic coverage than offered by the 27K platform. Using the Infinium HumanMethylation450 Beadchip (450K; Illumina Inc.), which measures CpG methylation at > 470,000 CpGs, we evaluated the relationship between maternal smoking and DNA methylation in 1,062 infant cord blood samples from a birth cohort in Norway. We assessed maternal smoking objectively by measuring cotinine, a sensitive biomarker, in maternal plasma samples, and replicated our findings in an independent birth cohort study from the U.S. To our knowledge, this is the largest human study of any *in utero* exposure in relation to DNA methylation at birth using the 450K platform with improved epigenome-wide coverage.

## Methods

Participants in the current analysis were selected from a substudy of the Norwegian Mother and Child Cohort Study (MoBa) (Magnus et al. 2006; Ronningen et al. 2006) that evaluated the association between maternal plasma folate during pregnancy and childhood asthma status at 3 years of age (Håberg et al. 2011). We analyzed 1,062 participants of this study who had available cord blood samples and nonmissing data for maternal cotinine and covariates. Umbilical cord blood samples were collected at birth and frozen at –80°C. All biological material was obtained from the biobank of the MoBa study (Ronningen et al. 2006). The MoBa study has been approved by the Regional Committee for Ethics in Medical Research, the Norwegian Data Inspectorate, and the Institutional Review Board of the National Institute of Environmental Health Sciences, and written informed consent was provided by all participants. Participants in the replication analysis were part of the Newborn Epigenetics Study (NEST) and were recruited from prenatal clinics in Durham, North Carolina (Hoyo et al. 2011; Murphy et al. 2012). We selected cord blood DNA samples from 18 newborns born to mothers who reported smoking during pregnancy and 18 whose mothers reported no smoking, all Caucasians. The NEST study has been approved by the Duke University Institutional Review Board, and written informed consent was provided by all participants.

Bisulfite conversion was performed using the EZ-96 (MoBa samples) or EZ (NEST samples) DNA Methylation kit (Zymo Research Corporation, Irvine, CA) according to manufacturer instructions. We measured methylation at 485,577 CpGs in cord blood using Illumina’s Infinium HumanMethylation450 BeadChip (Bibikova et al. 2011; Sandoval et al. 2011). Illumina’s GenomeStudio® Methylation module version 1.0 (Illumina Inc.) was used to calculate the methylation level at each CpG as the beta-value [β = intensity of the methylated allele (M) / (intensity of the unmethylated allele (U) + intensity of the methylated allele (M) + 100)] (Bibikova et al. 2011). Beta-values were then transformed to obtain the log ratio, defined as log[β/(1 – β)]. We report the detection *p*-value for each beta, which represents a statistical test for the difference between the signal for a given probe and background (the average for all negative controls).

Maternal plasma cotinine concentrations in the MoBa samples were measured using liquid chromatography–tandem mass spectrometry (Midttun et al. 2009). With guidance from a previous study with data on circulating cotinine in pregnant women (Shaw et al. 2009), we created four categories of exposure (undetectable: ≤ 0 nmol/L; low: > 0–56.8 nmol/L; moderate: > 56.8–388 nmol/L; high: > 388 nmol/L), where 56.8 nmol/L indicates active maternal smoking and participants with levels > 56.8 nmol/L were categorized at the median value for this group (388 nmol/L). Smoking in the NEST samples was assessed by maternal self-report of smoking during pregnancy and verified by medical records.

Quality control protocols are described in detail in Supplemental Material, pp. 3–5 (http://dx.doi.org/10.1289/ehp.1205412). After quality control, 1,062 MoBa subjects and 473,844 CpGs were analyzed. We examined the association between maternal plasma cotinine and methylation in cord blood at each CpG using robust linear regression to account for any potential outliers or heteroskedasticity in the data (Fox and Weisberg 2011). We evaluated maternal age (continuous variable), maternal education (< high school, high school, some college, and ≥ 4 years of college as the referent group), maternal pre-pregnancy body mass index (< 18.5, 18.5 to < 25, 25 to < 30, ≥ 30), maternal physical activity during pregnancy (none, moderate, vigorous), maternal plasma folate (log-transformed continuous variable), parity (0 as the referent group, 1, 2, and ≥ 3), and sex of the child for associations with maternal plasma cotinine. The final model included variables that were associated with cotinine (*p* < 0.1) and plausibly related to methylation levels, specifically maternal age, maternal education, and parity. Because our study was a subset of that of Håberg et al. (2011), where cotinine was measured among asthmatic children in a later analytic batch, we adjusted for childhood asthma status at 3 years of age although this made little difference in the results. Overall, the crude and adjusted results were extremely similar. We applied Bonferroni correction, adjusting the level of significance from 0.05 to 1.06 × 10^–7^. Additional adjustment for principal components to address potential population structure (Rakyan et al. 2011) was explored but not retained because it did not influence the results.

All NEST samples and CpGs passed quality control. We used unadjusted linear regression models to examine the association between maternal smoking during pregnancy and methylation in cord blood at each of the 26 CpGs that were significantly associated with plasma cotinine (*p* < 1.06 × 10^–7^) in the MoBa population, and we calculated a one-sided *p*-value for each CpG. We applied Bonferroni correction for 26 tests, which adjusted the level of significance to 0.0019. We used the Kolmogorov test to test the null hypothesis that the *p*-values are uniformly distributed on (0,1) against the alternative that the *p*-values were stochastically smaller than uniform distribution on (0,1). Because CpGs corresponding to the same gene may not be independent, and because some genes were represented by multiple CpGs, for such genes we chose the CpG that had the largest *p*-value when using the Kolmogorov test, thus making the Kolmogorov test more conservative against the alternative hypothesis.

To assess the potential impact of variation according to white blood cell subtype, we measured DNA methylation using the 450K platform in 21 cord blood samples collected at the same facilities as the NEST samples that had been separated while fresh into mononuclear cells (MN) and polymorphonuclear cells (PM) using Lympholyte®-poly (Cedarlane Laboratories Limited, Hornby, Ontario). We used a paired *t*-test to evaluate differential methylation between PM and MN cell types for our top 26 CpGs.

To compare our results with those from other studies, we employed a single CpG lookup approach where we looked at the result in our data for the CpG of interest and did not correct for multiple comparisons. A CpG with a *p*-value < 0.05 was considered to be statistically significant. All statistical analyses were performed using R (R Development Core Team 2010) and Bioconductor (Gentleman et al. 2004) packages.

## Results

**Figure 1:**
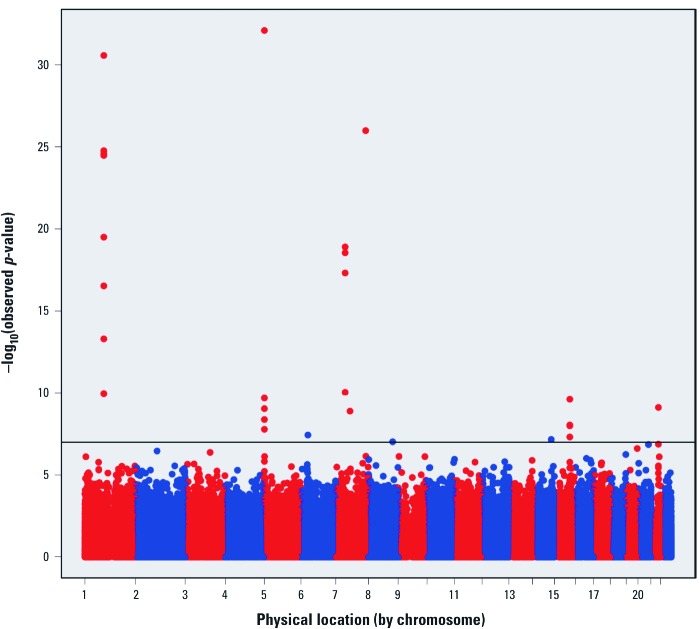
Epigenome-wide association between maternal cotinine and methyla­tion of 473,844 CpGs ­measured in cord blood from the MoBa cohort. Twenty-six CpGs (10 genes) reached Bonferroni-corrected statistical significance (p < 1.06 × 10^–7^, represented by the horizontal line). Red and blue alternating colors are used to distinguish between chromosomes.

*Epigenome-wide analysis.* We plotted the –log10(*p*-values) from the robust linear regression for 473,844 CpGs across the genome in 1,062 cord blood samples of participants in the MoBa study (Figure 1). The mean (± SD) age of study participants was 29.5 ± 4.3 years, 12.8% had plasma cotinine levels consistent with active smoking (Shaw et al. 2009), and 11.7% reported current smoking during pregnancy (Table 1). The methylation intensities showed bimodal distribution when displayed across all probes [see Supplemental Material, Figure S1A,B for beta and log-ratio values, respectively (http://dx.doi.org/10.1289/ehp.1205412)] but approximately normal distribution for most CpGs when they were considered individually (see Supplemental Material, Figure S1C,D).

**Table 1:** Descriptive characteristics of the MoBa study population (*n* = 1,062).

Characteristic	*n* (%)
Maternal cotinine (nmol/L)a
Undetectable (0)	736 (69.3)
Low (> 0–56.8)	190 (17.9)
Moderate (> 56.8–388)	68 (6.4)
High (> 388)	68 (6.4)
Self-reported maternal smoking
Never	525 (49.4)
Former (stopped before pregnancy)	233 (21.9)
Stopped during pregnancy	180 (16.9)
Sometimes or daily	124 (11.7)
Child sex
Male	566 (53.3)
Female	496 (46.7)
Maternal education
< High school	78 (7.3)
High school	343 (32.3)
Some college	471 (44.4)
≥ 4 years of college	170 (16.0)
Parity
0	447 (42.1)
1	435 (41.0)
2	137 (12.9)
≥ 3	43 (4.0)
aMaternal cotinine measured in plasma at approximately gestational week 18. Cotinine values > 56.8 nmol/L are consistent with active smoking.		

Using conservative Bonferroni correction for 473,844 tests, we observed epigenome-wide statistically significant associations (*p*-value < 1.06 × 10^–7^) between maternal plasma cotinine and DNA methylation for 26 CpGs mapped to 10 genes in both unadjusted and covariate-adjusted analyses (Figure 1, Table 2). Four genes included at least four Bonferroni-significant CpGs. Among the 26 CpGs, 8 were within the coding region of growth factor independent 1 transcription repressor (*GFI1*) on chromosome 1; 4 were within the coding region of aryl-hydrocarbon receptor repressor (*AHRR*) on chromosome 5; 4 were in a region upstream of cytochrome P450 isoform *CYP1A1* on chromosome 15; and 4 were within the coding region of myosin 1G (*MYO1G*).

**Table 2:** Differential methyla­tion in cord blood DNA in relation to maternal cotinine in the MoBa study population: CpGs with Bonferroni-corrected statistical significance (p < 1.06 × 10^–7^), sorted by chromosome and position.

Distance to gene^b^	Unadjusted					Adjusted^f^					Median methylation by cotinine category^h^			
	Chr^a^	Gene	CpG	Position^c^	Coef^d^	SE^e^	*p*-Value	Coef	SE	*p*-Value	Rank^g^	Undetectable	Low	Medium	High
1	GFI1	3688	cg10399789	92945668	–0.07	0.01	4.08E-13	–0.065	0.010	1.10E-10	14	0.759	0.755	0.727	0.716
1	GFI1	3224	cg09662411	92946132	–0.111	0.012	2.26E-20	–0.106	0.013	2.96E-17	11	0.730	0.733	0.669	0.654
1	GFI1	3169	cg06338710	92946187	–0.112	0.013	1.34E-18	–0.106	0.014	5.02E-14	12	0.801	0.800	0.754	0.733
1	GFI1	2656	cg18146737	92946700	–0.28	0.024	2.42E-30	–0.271	0.026	3.30E-25	6	0.877	0.875	0.771	0.738
1	GFI1	2531	cg12876356	92946825	–0.182	0.016	2.29E-30	–0.176	0.017	1.70E-25	4	0.731	0.732	0.627	0.605
1	GFI1	2321	cg18316974	92947035	–0.243	0.024	6.43E-24	–0.238	0.026	3.16E-20	7	0.921	0.923	0.856	0.841
1	GFI1	1768	cg09935388	92947588	–0.196	0.015	1.05E-38	–0.188	0.016	2.68E-31	2	0.708	0.707	0.580	0.564
1	GFI1	1395	cg14179389	92947961	–0.184	0.017	5.38E-28	–0.181	0.017	2.63E-25	5	0.242	0.246	0.154	0.158
5	AHRR	19617	cg23067299	323907	0.075	0.012	4.21E-10	0.072	0.012	4.12E-09	20	0.789	0.789	0.813	0.837
5	AHRR	64157	cg03991871	368447	–0.057	0.008	2.04E-11	–0.054	0.009	1.99E-10	15	0.841	0.839	0.820	0.818
5	AHRR	69088	cg05575921	373378	–0.202	0.015	2.85E-39	–0.198	0.017	8.03E-33	1	0.883	0.874	0.829	0.784
5	AHRR	95070	cg21161138	399360	–0.045	0.007	1.52E-11	–0.043	0.007	8.91E-10	18	0.718	0.715	0.701	0.679
6	HLA-DPB2	11549	cg11715943	33091841	–0.053	0.009	1.00E-08	–0.054	0.010	3.63E-08	23	0.842	0.833	0.824	0.820
7	MYO1G	16417	cg19089201	45002287	0.083	0.013	3.22E-10	0.088	0.014	9.13E-11	13	0.925	0.926	0.932	0.944
7	MYO1G	16218	cg22132788	45002486	0.18	0.021	1.98E-18	0.184	0.021	4.82E-18	10	0.932	0.935	0.951	0.966
7	MYO1G	15968	cg04180046	45002736	0.073	0.008	8.76E-20	0.076	0.008	2.85E-19	9	0.441	0.446	0.484	0.508
7	MYO1G	15785	cg12803068	45002919	0.145	0.016	8.51E-19	0.149	0.016	1.25E-19	8	0.713	0.721	0.774	0.813
7	ENSG00000225718	198306	cg04598670	68697651	–0.063	0.009	1.29E-11	–0.061	0.010	1.27E-09	19	0.623	0.607	0.597	0.574
7	CNTNAP2	854	cg25949550	145814306	–0.075	0.007	4.15E-30	–0.073	0.007	1.02E-26	3	0.113	0.109	0.097	0.092
8	EXT1	–33821	cg03346806	119157879	–0.038	0.007	3.08E-08	–0.039	0.007	9.34E-08	26	0.801	0.795	0.793	0.779
14	TTC7B	274756	cg18655025	91008005	–0.041	0.007	2.07E-08	–0.042	0.008	6.76E-08	25	0.854	0.847	0.841	0.836
15	CYP1A1	–1266	cg05549655	75019143	0.064	0.01	2.96E-10	0.065	0.010	2.38E-10	16	0.189	0.188	0.221	0.226
15	CYP1A1	–1374	cg22549041	75019251	0.096	0.016	4.52E-09	0.098	0.017	8.88E-09	21	0.385	0.379	0.414	0.475
15	CYP1A1	–1406	cg11924019	75019283	0.044	0.008	2.62E-08	0.044	0.008	4.78E-08	24	0.434	0.430	0.457	0.475
15	CYP1A1	–1425	cg18092474	75019302	0.066	0.012	1.10E-08	0.068	0.012	9.95E-09	22	0.510	0.504	0.549	0.573
21	RUNX1	1746	cg12477880	36259241	0.159	0.026	1.02E-09	0.163	0.026	7.55E-10	17	0.088	0.102	0.110	0.158
aChromosome. bDistance from CpG to transcription start site of the nearest gene. cChromosomal position based on NCBI human reference genome assembly Build 37.3. dRegression coefficient. eStandard error for regression coefficient. fAdjusted for maternal age, maternal education, parity, and asthma. gRank order based on the adjusted p-value. hMaternal plasma cotinine (nmol/L) measured around gestational week 18 (undetectable: ≤ 0; low: > 0–56.8; moderate: > 56.8–388; high: > 388). Values > 56.8 nmol/L indicate active smoking.																								

Methylation levels of *AHRR* cg05575921, the CpG with the smallest *p*-value in the analysis, decreased with cotinine in a dose-dependent manner, and the trend was statistically significant [see Supplemental Material, Figure S2, Jonkheere–Terpstra trend test *p* < 2.2 × 10^–16^ (http://dx.doi.org/10.1289/ehp.1205412)]. All other statistically significant *AHRR* CpGs (see Supplemental Material, Figure S3A) had lower methylation with increasing cotinine levels except for cg23067299, which is upstream of the other significant *AHRR* CpGs and had higher methylation with increasing cotinine (see Supplemental Material, Table S1). Conversely, *CYP1A1* cg05549655 (*p* = 2.38 × 10^–10^) and other statistically significant *CYP1A1* CpGs (see Supplemental Material, Figure S3B), had higher methylation with increasing levels of cotinine. As with *AHRR*, cotinine was inversely related to methylation for *GFI1* cg09935388 (*p* = 2.68 × 10^–31^) and other statistically significant *GFI1* CpGs. All four statistically significant CpGs in *MYO1G* had higher methylation with increasing levels of cotinine (Table 2). Other genes in which methylation was associated with cotinine levels at epigenome-wide statistical significance are *HLA-DPB2*, *ENSG00000225718, TTC7B, CNTNAP2, EXT1,* and *RUNX1* (Table 2). Coefficients, *p*-values, locations, and other information for the 100 most statistically significant CpGs are provided in Supplemental Material Table S1.

We obtained similar DNA methylation differences when the metric of exposure was maternal self-report of smoking during pregnancy rather than measured maternal cotinine levels. Specifically, self-reported maternal smoking was related to lower methylation of *AHRR* CpGs cg03991871, cg05575921, and cg21161138, with similar regression coefficients as in the cotinine analysis, and was epigenome-wide statistically significant for cg05575921 [regression coefficient (coef) = –0.131, SE = 0.017, *p* = 2.40× 10^–15^]. Self-reported maternal smoking was associated with higher methylation of the *CYP1A1* CpGs shown in Table 2 but did not achieve epigenome-wide statistical significance (e.g., cg05549655: coef = 0.039, SE = 0.010, *p* = 1.72 × 10^–4^). Self-reported maternal smoking was associated with epigenome-wide statistically significant lower methylation of seven of the eight *GFI1* CpGs that had significant (*p* < 1.06 × 10^–7^) inverse associations with maternal cotinine. Higher methylation of the four *MYO1G* CpGs statistically significantly associated with maternal cotinine was observed with maternal smoking but did not reach epigenome-wide statistical significance.

**Figure 2:**
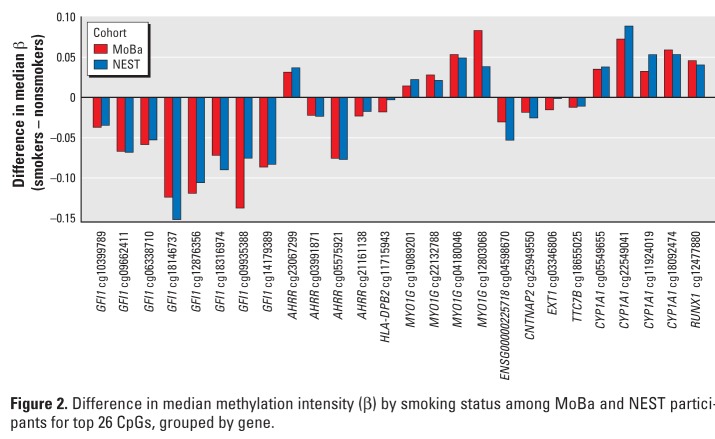
Difference in median methyla­tion intensity (β) by smoking status among MoBa and NEST participants for top 26 CpGs, grouped by gene.

*Replication analysis.* All twenty-six CpGs showing epigenome-wide statistical significance for the association between plasma cotinine and methylation in MoBa were followed up for replication analysis in cord blood DNA samples from offspring of 18 mothers who reported smoking during pregnancy and 18 mothers who denied smoking during pregnancy from the NEST study (Table 3). The direction of effect (differential methylation by smoking status) in the NEST replication study was consistent with the discovery study (MoBa) for all 26 CpGs, and the magnitudes of the differences between smokers and nonsmokers in NEST and those between women with plasma cotinine > 56.8 nmol/L versus ≤ 56.8 nmol/L in MoBa were very similar (Table 3, Figure 2, Spearman’s correlation coefficient = 0.965). The most statistically significant association in both MoBa and NEST data was observed for *AHRR* cg05575921 with lower methylation for smokers compared with nonsmokers (Table 3). In the NEST study, a total of five CpGs (*AHRR* cg05575921, *CYP1A1* cg05549655 and cg11924019, and *GFI1* cg09935388 and cg12876356) reached statistical significance after strict Bonferroni correction for 26 tests (*p* < 0.0019), despite the much smaller sample size of the replication study (18 newborns of smoking mothers compared with 18 newborns of nonsmoking mothers), and a total of 21 of the 26 CpGs gave a *p*-value < 0.05 (Table 3). The Kolmogorov test showed that the replication *p*-values were systematically smaller than would be expected by chance (*p* < 0.00011). This suggests that it is exceedingly unlikely that the replication findings are false positives and confirms the high degree of replication that we observed.

**Table 3:** Replication results from the NEST cohort for the 26 CpGs reaching epigenome-wide Bonferroni-corrected statistical significance (p < 1.06 × 10^–7^) in the MoBa cohort, and median methyla­tion differences by maternal smoking for MoBa and NEST cohorts, sorted by NEST *p*-value

NEST *p*-value^a^	Percent difference in median methylation (smokers – nonsmokers)		
	Chromosome	Gene	CpG	MoBab	NESTc
5	AHRR	cg05575921	0.0003*	–7.5	–7.7
15	CYP1A1	cg05549655	0.0006*	3.5	3.8
15	CYP1A1	cg11924019	0.0008*	3.2	5.3
1	GFI1	cg09935388	0.0012*	–13.7	–7.5
1	GFI1	cg12876356	0.0015*	–11.9	–10.5
1	GFI1	cg18316974	0.0023	–7.1	–9.0
1	GFI1	cg09662411	0.0023	–6.6	–6.8
7	CNTNAP2	cg25949550	0.0025	–1.8	–2.5
1	GFI1	cg06338710	0.0026	–5.8	–5.2
7	MYO1G	cg04180046	0.0027	5.3	4.9
7	ENSG00000225718	cg04598670	0.0036	–3.0	–5.3
5	AHRR	cg23067299	0.0036	3.2	3.7
1	GFI1	cg18146737	0.0037	–12.3	–15.1
7	MYO1G	cg12803068	0.0041	8.3	3.8
1	GFI1	cg14179389	0.0043	–8.6	–8.2
15	CYP1A1	cg22549041	0.0044	7.2	8.9
15	CYP1A1	cg18092474	0.0044	5.9	5.3
7	MYO1G	cg19089201	0.0092	1.4	2.2
7	MYO1G	cg22132788	0.0096	2.8	2.1
1	GFI1	cg10399789	0.0154	–3.7	–3.4
5	AHRR	cg21161138	0.0283	–2.3	–1.7
5	AHRR	cg03991871	0.0655	–2.2	–2.3
21	RUNX1	cg12477880	0.0850	4.6	4.0
8	EXT1	cg03346806	0.1248	–1.5	–0.1
14	TTC7B	cg18655025	0.2668	–1.2	–1.1
6	HLA-DPB2	cg11715943	0.7956	–1.8	–0.3
ap-Value from NEST replication linear regression model evaluating differential DNA methylation by maternal smoking during pregnancy. bMoBa maternal smoking status determined by gestational week 18 cotinine values > 56.8 nmol/L (smoker) or ≤ 56.8 nmol/L (nonsmoker). cNEST maternal smoking status determined by maternal self-report, verified by medical records. *Bonferroni-corrected statistically significant (p < 0.0019).										

The magnitudes of the differences in methylation between PM and MN cell types (Table S2) were much smaller than the differences in methylation between smokers and nonsmokers in both the MoBa and NEST study populations (Table 3) and were not statistically significantly different between cell types (using *p* < 0.0019 after Bonferroni correction for 26 tests) for the 5 replicated CpGs.

## Discussion

Our study of maternal smoking in relation to epigenome-wide DNA methylation in newborns in the MoBa cohort is the largest and most extensive that we know of. In addition to the large sample size, we used a highly reproducible platform that assesses methylation at > 470,000 individual CpGs, providing more comprehensive coverage of the epigenome than other studies of maternal smoking published to date. Further, we assessed maternal smoking with a sensitive assay for cotinine, a well-validated biomarker for tobacco smoke. We observed epigenome-wide statistically significant associations between maternal smoking in pregnancy, assessed by plasma cotinine levels and methylation in cord blood at 26 CpGs mapping to 10 genes in MoBa. In an independent birth cohort from the United States, the NEST study, we found a striking degree of replication for our findings. In the NEST replication population, the direction of differential methylation in relation to maternal smoking was consistent with the direction in relation to maternal plasma cotinine for all of the 26 CpGs that were significant (*p* < 1.06 × 10^–7^) in the discovery study. In addition, despite the more modest sample size of the replication set (18 newborns born to smoking mothers and 18 born to nonsmokers), estimates for 21 of 26 CpGs had *p*-values < 0.05. Five CpGs of the 26 met strict Bonferroni-corrected statistical significance in the replication study (*p* < 0.0019): two in *CYP1A1* and one in *AHRR,* genes known to be involved in the detoxification of compounds from tobacco smoke via the aryl hydrocarbon receptor (AhR) signaling pathway; and two CpGs in *GFI1*, a gene that has not previously been implicated in responses to tobacco smoke.

Our most statistically significant finding in both the replication and discovery analyses was lower methylation with higher levels of self-reported or cotinine-based evidence of maternal smoking at cg05575921 in *AHRR*. Remarkably, a recent study in adults, using the same 450K platform, showed lower methylation at this same CpG (cg05575921) in smokers compared with nonsmokers at epigenome-wide statistical significance (Monick et al. 2012). That study observed lower methylation in smokers at this CpG for both lymphoblasts and pulmonary alveolar macrophages (Monick et al. 2012). The authors also studied the functional implications of this methylation change and found that methylation at *AHRR* cg05575921 was associated with *AHRR* expression. Thus our data show that a methylation change found in adult smokers and implicated as functionally important in *AHRR*, a gene involved in a key pathway of response to tobacco smoke components, is already present at birth in newborns due to maternal smoking in pregnancy.

Our findings for genes in the AhR pathway make sense biologically; the pathway is known to mediate the effects of toxicants such as polycyclic aromatic hydrocarbons (PAH) in tobacco smoke. PAH bind to AhR causing its translocation to the nucleus and the formation of a heterodimer with the AhR nuclear transporter. This complex binds DNA regulatory sequences, termed xenobiotic response elements (XREs), and initiates the expression of *CYP1A1* and other genes involved in detoxification of these chemicals (Nguyen and Bradfield 2008). The AhR repressor (AHRR) acts as a negative regulator of AhR activity, suppressing *CYP1A1* transcription (Harper et al. 2006). In our study, maternal smoking, assessed objectively by cotinine, displayed a dose-dependent association with lower methylation of *AHRR* CpGs and higher methylation of *CYP1A1* CpGs in cord blood. The contrasting effects of maternal smoking during pregnancy on methylation at CpGs in *AHRR* and *CYP1A1* are notable because of the opposing function these genes have in the AhR pathway (Kawajiri and Fujii-Kuriyama 2007).

Although the role of the AhR pathway in response to toxicants is well known, there is increasing identification of the importance of this pathway in the regulation of other processes, such as immune function (Lawrence and Sherr 2012). In addition, AhR has also recently been found to play a crucial role in regulating cigarette smoke extract–induced apoptosis in fibroblasts (lung and embryonic) and lung epithelial cells in culture from the mouse (Rico de Souza et al. 2011).

We also replicated our novel findings for *GFI1,* which has not previously been implicated in response to tobacco smoke. *GFI1* plays an essential role in diverse developmental processes including hematopoiesis and the development of the inner ear and pulmonary neuroendocrine cells (Duan et al. 2005; Khandanpour et al. 2011). *GFI1* influences numerous cellular events such as proliferation, apoptosis, differentiation, lineage decisions, and oncogenesis (Jafar-Nejad and Bellen 2004). This gene is part of a complex that enables histone modifications and may also control alternative pre-mRNA splicing (Moroy and Khandanpour 2011). Given the pivotal involvement of *GFI1* in fundamental development processes, a role in diverse effects of maternal smoking on the offspring is biologically plausible.

Although our findings for *RUNX1* did not meet strict Bonferroni statistical significance in the replication population (NEST *p* > 0.00019), there were four *RUNX1* CpGs in the top 100 results in the MoBa discovery population [see Supplemental Material, Table S1 (http://dx.doi.org/10.1289/ehp.1205412)]. *RUNX1* (also known as *AML1*) is involved in the development of normal hematopoiesis as well as leukemia (Kumano and Kurokawa 2010). Of note, *RUNX1*, AhR, and *GFI1* are all involved in the regulation of hematopoietic stem cells (Boitano et al. 2010; Khandanpour et al. 2011; Oshima et al. 2011), suggesting the possibility that cross-talk between these genes may impact smoking-related health outcomes in the offspring.

Correlation of DNA methylation at CpGs within the same gene may contribute to the finding of genes with multiple significant CpGs in our analyses. However, if CpGs are not truly independent, then using strict Bonferroni correction for multiple testing, which assumes independent tests, is quite conservative. This adds support for the results that surpassed this strict threshold, particularly the five CpGs with corrected statistical significance in both the discovery and replication populations.

Cotinine, the biomarker of smoking, was not measured among pregnant women in the replication (NEST) study, so we used maternal self-report of smoking on questionnaires that was consistent with medical records of smoking. Among our MoBa study participants, 8 of the 136 women (5.9%) with cotinine values consistent with active smoking (≥ 56.8 nmol/L) reported that they did not smoke during pregnancy. A study of U.S. reproductive age women using NHANES (National Health and Nutrition Examination Survey) data indicates that U.S. pregnant women also underreport smoking (Dietz et al. 2011). Thus, we expect that some of the NEST participants classified as nonsmokers might actually have been smokers. However, this type of misclassification should lead to a bias of estimates toward the null, making our replication estimates more conservative than would be expected if smokers had reported their exposure with 100% accuracy.

The 450K Beadchip offers greatly improved genomic coverage over the earlier 27K platform. The 450K content includes 99% of RefSeq genes with multiple probes per gene, 96% of CpG islands from the UCSC database (http://genome.ucsc.edu/), CpG island shores, and additional content selected from whole-genome bisulfite sequencing data and input from DNA methylation experts (Bibikova et al. 2011). None of the 26 CpGs with epigenome-wide significance in our study were present on the 27K platform. A study using the 27K platform observed differences in DNA methylation associated with smoking status in adults at CpG cg03636183 in *F2RL3*, a gene associated with cardiovascular disease (Breitling et al. 2011). Employing a single CpG look-up approach (one CpG evaluated, uncorrected for multiple testing), our data provide support for an association between maternal smoking during pregnancy and cord blood DNA methylation at this CpG (coef = –0.020, se = 0.009, *p* = 0.016). Another recent study of 27K methylation and adult smoking identified cg19859270 in *GPR15* (Wan et al. 2012). Our single look-up approach for this CpG did not provide supporting results (coef = –0.010, SE = 0.007, *p* = 0.181).

Maternal smoking during pregnancy has been associated with CpG-specific differential DNA methylation in placental tissue using the 27K Beadchip (Suter et al. 2011), but we found no overlap between the top hits from that study and the top hits in our study using the 450K Beadchip to measure methylation in newborn cord blood samples. In addition to the limitations of comparing the 27K and 450K platforms (the top 26 CpGs from our study were not covered on the 27K platform), it is likely that altered methylation in response to tobacco smoke exposure is different in placental tissue and newborn cord blood.

We measured DNA methylation in whole cord blood samples. Because hematopoietic differentiation and methylation status of differentially methylated regions (DMRs) are highly coordinated (Schmidl et al. 2009), significant shifts in cell type pools in the blood should be accompanied by shifts in methylation at dozens, if not hundreds, of cell type–specific DMRs. If our findings were simply a reflection of maternal smoking influencing shifts in cell types, we would expect to find many differentially methylated genes, which we did not. Instead, only 10 genes had differences in methylation levels related to cotinine in our data.

Notably, the recent paper of Monick et al. (2012) supports the notion that unmeasured confounding by cell type does not explain the altered methylation status that we observed in relation to smoking. In that paper, smoking-induced alteration of *AHRR* CpGs (including our top CpG) was seen in both B lymphoblastoid cells and in an independently collected sample of alveolar macrophage cells collected from bronchial lavage. Thus, Monick et al. identified smoking-induced signals across two distinct cell types, which strengthens our replicated whole blood findings.

Although the above evidence suggests that our results are not confounded by cell type, we directly addressed the potential impact of differential cell counts by measuring epigenome-wide DNA methylation, using the 450K assay, in 21 cord blood samples that had been separated, while fresh, into the two major cell pools, PM cells and MN cells. In these 21 paired samples differences in methylation by cell type were very small. These small differences were statistically significant (*p* < 0.0019) for 3 of the top 26 CpGs associated with maternal plasma cotinine in MoBa, but these CpGs were not significantly associated with maternal smoking in the NEST population. Furthermore, the magnitude of the difference in median methylation between PM and MN cell types was much smaller than the difference in median methylation between smokers and nonsmokers in both the MoBa and NEST study populations. For the percent difference in median methylation by cell type, the maximum was 3.1% and the mean was 1.0%. In contrast, for the percent difference in median methylation between smokers and non-smokers measured in whole blood, which is a mixture of these two major cell types (PM and MN), the maximum was 13.7% (MoBa) and 15.1% (NEST), and the mean was 5.3% (MoBa) and 5.0% (NEST). For our top CpG *AHRR* cg05575921, the percent difference in median methylation was 0.31% by cell type compared with 7.52% for smokers compared with nonsmokers in MoBa (7.67% in NEST). Thus, the differences in methylation between these two major cell pools are much smaller than the differences in methylation by smoking that we observed in whole blood, suggesting that confounding by cell type is unlikely to explain our findings of differential methylation related to smoking.

It is possible that differences in methylation may exist in more refined subclassifications of cell types that we did not specifically examine. But again, our top finding for *AHRR* cg05575921 due to maternal smoking was also reported in adult smokers in two different cell types—alveolar lung macrophage DNA and lymphoblast DNA (Monick et al. 2012). These various lines of evidence give strong support for the conclusion that our replicated findings are not explained by effects of maternal smoking on the relative prevalence of white blood cell subtypes that differ with regard to CpG methylation.

## Conclusions

*In utero* exposure to maternal smoking is an important risk factor for numerous adverse outcomes in children and adults. With a hypothesis-free epigenome-wide screen with replication in a second population, we observed strong evidence that maternal smoking during pregnancy is associated with cord blood methylation of genes in the AhR signaling pathway—important for the detoxification of xenobiotics in tobacco smoke—and methylation of a novel gene not previously implicated in response to tobacco smoke that is involved in fundamental developmental processes. These results suggest that epigenetic mechanisms reflected by DNA methylation may underlie some of the well-documented impacts of maternal smoking on offspring. Our identification of differential methylation in genes known to be involved in the response to tobacco-related compounds, in addition to a novel gene, demonstrates the value of using this approach to elucidate the epigenetic effects of *in utero* exposures.

## Supplemental Material

(627 KB) PDF
