# Supplementary material for: 450K Epigenome-Wide Scan Identifies Differential DNA Methylation in Newborns Related to Maternal Smoking during Pregnancy
Source: Environ Health Perspect. 2012 Jul 31;120(10):1425–31. doi: 10.1289/ehp.1205412 (PMC3491949; doi:10.1289/ehp.1205412)
Supplement: (627 KB) PDF [file ehp.1205412.s001.pdf]

Supplemental Material

450K Epigenome-Wide Scan Identifies Differential DNA Methylation in Newborns Related to Maternal Smoking During Pregnancy

Bonnie R. Joubert [1], Siri E. Håberg [2], Roy M. Nilsen [3], Xuting Wang [1], Stein E. Vollset [2,4], Susan K. Murphy [5], Zhiqing Huang [5], Cathrine Hoyo [5],

Øivind Midttun [6], Lea A. Cupul-Uicab [1], Per M Ueland [4], Michael C. Wu [7], Wenche Nystad [2], Douglas A. Bell [1], Shyamal D. Peddada [1], Stephanie J. London [1]*

[1] Division of Intramural Research, National Institute of Environmental Health Sciences, National Institutes of Health, Department of Health and Human Services, Research Triangle Park, North Carolina; [2] Norwegian Institute of Public Health, Oslo, Norway; [3] Haukeland University Hospital, Bergen, Norway; [4] University of Bergen, Bergen, Norway; [5] Duke University School of Medicine, Durham NC; [6] Bevital A/S, Laboratoriebygget, Bergen, Norway; [7] University of North Carolina, Chapel Hill, NC

Table of Contents

Supplemental Text, pages 3-5

Table S1, pages 6-8

Table S2, page 9

Figure S1, page 10

Figure S2, page 11

Figure S3, pages 12-13

Supplemental Text

Quality Control

Bisulfite conversion for the MoBa samples was evaluated according to methods previously described (Bibikova et al. 2011). Additionally, we included 28 blind replicate samples (14 study subjects run in duplicate, included on each plate), 26 plate control samples provided by Illumina (DNA from two cells lines run on each of the 13, 96-well plates), 25 plate control samples provided by us [DNA from 2 control individuals on each of 12 plates (1 on each half plate), 1 on the 13th plate] and 8 samples prepared from mixing methylated and non-methylated DNA, described as follows. Human HCT116 DKO Methylated DNA (Cat# D5014-2) and human HCT116 DKO non-methylated DNA (Cat# D5014-1) were purchased from Zymo Research (Irvine, CA). The fully methylated DNA was mixed with non-methylated DNA to provide a series of methylation controls (10%, 35%, 60%, and 85% methylated) to be included on the first and twelfth plates.

We received from Illumina (San Diego, CA), data for a total of 1,204 samples. Samples with an average detection p-value across all probes of less than 0.05 and/or indicated by Illumina to have failed (N=49) were omitted from further analysis along with 1 sample erroneously included in the dataset. Multidimensional scaling (MDS) plots were used to evaluate gender outliers based on chromosome X data, where males and females separated into two distinct clusters. Samples separating into erroneous clusters (males in female cluster or females in male cluster) or not belonging to a distinct cluster were omitted (N=13). Blind duplicate samples were highly correlated (Spearman rho = 0.997) and the mean difference in beta was 0.0043 (standard error = 0.00012). For the 14 blind duplicate pairs, results from one of the two samples in each

pair was selected at random to retain in the dataset and the other was omitted from further analysis. CpGs with missing chromosome data (N=65, mostly control probes), missing more than 10% of data across individuals (N=20), or on chromosome X (N=11,232) or Y (N=416) were omitted, resulting in 473,844 probes for analysis.

The laboratory analysis plan was designed to exclude batch effects. All samples were run with a single set of reagents on a single machine at Illumina, Inc. (San Diego, CA). Bisulfite conversion and methylation measurements including reruns were performed in March 2011. Variables representing the chip (12 samples), chip set (four contiguous chips or half of a plate), and plate (96 samples) were included as covariates in statistical models to evaluate potential confounding. In addition, the distributions of beta and logratio values were compared across chips, chip sets and plates. We found that chip, chip set and plate were not appreciable sources of variability.

The NEST data quality control followed a similar protocol as described above. In addition to the 18 smokers (9 males, 9 females) and 18 non-smokers (9 males, 9 females), eight plate control samples and three samples representing 10%, 50%, and 85% methylation were included on a single plate.

It is possible that SNPs at or near CpGs could influence methylation intensities and thereby the associations we observed. We searched online databases to determine the presence of an underlying SNP for the top 105 most statistically significant CpGs. Information was obtained for SNPs with minor allele frequency ≥ 5% in the CEU (Utah residents with Northern and Western European ancestry) population, curated by 1000G projects (http://www.1000genomes.org/, 06/2011 release, 87 individuals), HapMap project (http://hapmap.ncbi.nlm.nih.gov/, release 28, 8/2010, 174 individuals), and dbSNP

(http://www.ncbi.nlm.nih.gov/projects/SNP/, build 134, 8/2011, 116 individuals). We found 3 probes with SNPs within CpGs and removed them from the top 105 findings (rs77990586 in *WBSCR17* cg20370581, rs115039881 in cg00531338 not specific to a gene, and rs6869832 in *AHRR* cg23576855).

References

Bibikova M, Barnes B, Tsan C, Ho V, Klotzle B, Le JM, et al. 2011. High density DNA methylation array with single CpG site resolution. Genomics.

Supplemental Material, Table S1. Differential methylation in cord blood DNA in relation to maternal cotinine, top 100 most significant CpGs, sorted by chromosome, position

Chr[a]	Gene	Distance[b]	Location[c]	CpG	Position[d]	Coef[e]	SE[f]	p-value*
1	*AJAP1*	20974	BODY	cg26435172	4736126	-0.033	0.007	7.66E-07
1	*WDR78*	734	BODY	cg00044354	67389884	-0.045	0.009	1.65E-06
1	*GNG12*	-338	TSS	cg25189904	68299493	-0.031	0.007	4.96E-06
1	*GNG12*	-356	TSS	cg26764244	68299511	-0.034	0.007	4.47E-06
1	*GFI1*	3688	BODY	cg10399789	92945668	-0.065	0.010	1.10E-10*
1	*GFI1*	3224	BODY	cg09662411	92946132	-0.106	0.013	2.96E-17*
1	*GFI1*	3217	BODY	cg06338710	92946187	-0.106	0.014	5.02E-14*
1	*GFI1*	2704	BODY	cg18146737	92946700	-0.271	0.026	3.30E-25*
1	*GFI1*	2531	BODY	cg12876356	92946825	-0.176	0.017	1.70E-25*
1	*GFI1*	2321	BODY	cg18316974	92947035	-0.238	0.026	3.16E-20*
1	*GFI1*	1768	BODY	cg09935388	92947588	-0.188	0.016	2.68E-31*
1	*GFI1*	1443	BODY	cg14179389	92947961	-0.181	0.017	2.63E-25*
1	*PBX1*	27680	BODY	cg07353489	164556276	-0.032	0.007	4.33E-06
1	*ENSG00000198216*	-2747	TSS	cg08943293	181449699	-0.029	0.006	2.90E-06
1	*MFSD4*	-2879	TSS	cg06827850	205535232	-0.030	0.007	4.16E-06
1	*GALNT2*	207374	BODY	cg11527913	230410329	-0.052	0.011	5.76E-06
1	*KIF26B*	180355	BODY	cg15137445	245498689	-0.027	0.006	2.80E-06
1	*OR14I1*	444	BODY	cg01165362	248845209	-0.037	0.008	5.74E-06
2	*ENSG00000232835*	-5198	TSS	cg14556323	5701409	-0.028	0.006	3.61E-06
2	*XDH*	78080	BODY	cg14654795	31559579	-0.033	0.007	5.72E-06
2	*EXOC6B*	222520	BODY	cg06108254	72830705	-0.030	0.007	4.96E-06
2	*LOC284998*	10689	BODY	cg18703066	105363536	-0.076	0.015	3.42E-07
2	*MYO1B*	20360	BODY	cg12738764	192130613	-0.033	0.007	2.76E-06
2	*ENSG00000228226*	-4057	TSS	cg20962638	229553401	-0.031	0.007	4.93E-06
2	*COPS8*	6478	BODY	cg05301057	238000561	-0.032	0.007	4.03E-06
2	*PP14571*	6909	BODY	cg18455650	241389208	-0.030	0.007	5.73E-06
3	*VGLL4*	11	TSS	cg18096987	11623873	-0.025	0.005	2.20E-06
3	*ENSG00000235886*	-99161	TSS	cg07746241	43920635	-0.027	0.006	2.10E-06
3	*ENSG00000243149*	-31477	TSS	cg22931622	65129155	-0.032	0.007	4.34E-06
3	*SEMA5B*	-1510	TSS	cg13428477	122748086	-0.041	0.008	4.16E-07
3	*SSR3*	-51017	TSS	cg26405475	156324038	0.042	0.009	5.66E-06
4	*ENSG00000249105*	-183313	TSS	cg07796335	59167549	-0.034	0.007	5.06E-06
5	*AHRR*	19569	BODY	cg23067299	323907	0.072	0.012	4.12E-09*
5	*AHRR*	64109	BODY	cg03991871	368447	-0.054	0.009	1.99E-10*
5	*AHRR*	64466	BODY	cg23916896	368804	-0.046	0.010	1.46E-06
5	*AHRR*	69088	BODY	cg05575921	373378	-0.198	0.017	8.03E-33*
5	*AHRR*	69088	BODY	cg21161138	399360	-0.043	0.007	8.91E-10*
5	*ENSG00000249201*	2484	BODY	cg01772854	1176225	-0.033	0.007	7.43E-07
5	*FSTL4*	86949	BODY	cg02378360	132861322	-0.031	0.007	3.04E-06
6	*RPP40*	2364	BODY	cg11942662	5001955	-0.030	0.007	4.26E-06
6	*OR12D2*	500	BODY	cg08862210	29364963	-0.036	0.008	3.33E-06
6	*TRIM15*	1441	BODY	cg23784132	30132471	-0.039	0.009	4.46E-06
6	*TRIM26*	21761	BODY	cg21531017	30159510	-0.030	0.006	2.28E-06

Supplemental Material, Table S1 (cont.). Differential methylation in cord blood DNA in relation to maternal cotinine, top 100 most significant CpGs, sorted by chromosome, position

Chr[a]	Gene	Distance[b]	Location[c]	CpG	Position[d]	Coef[e]	SE[f]	p-value*
6	*HLA-DPB2*	11549	BODY	cg11715943	33091841	-0.054	0.010	3.63E-08*
7	*SDK1*	7987	BODY	cg21005410	4177351	-0.029	0.006	3.26E-06
7	*MYO1G*	16465	BODY	cg19089201	45002287	0.088	0.014	9.13E-11*
7	*MYO1G*	16266	BODY	cg22132788	45002486	0.184	0.021	4.82E-18*
7	*MYO1G*	15968	BODY	cg04180046	45002736	0.076	0.008	2.85E-19*
7	*MYO1G*	15785	BODY	cg12803068	45002919	0.149	0.016	1.25E-19*
7	*ENSG00000225718*	39390	TSS	cg04598670	68697651	-0.061	0.010	1.27E-09*
7	*PODXL*	34603	BODY	cg21771679	131206773	-0.036	0.008	1.89E-06
7	*CNTNAP2*	854	BODY	cg25949550	145814306	-0.073	0.007	1.02E-26*
7	*CNTNAP2*	1090753	BODY	cg11207515	146904205	-0.041	0.008	7.14E-07
7	*PTPRN2*	277695	BODY	cg02356647	158102787	-0.037	0.008	3.24E-06
8	*ARHGEF10*	78878	BODY	cg26101086	1851026	-0.035	0.007	1.14E-06
8	*ERLIN2*	18617	BODY	cg26393977	37612713	-0.035	0.007	2.62E-06
8	*EXT1*	-33821	TSS	cg03346806	119157879	-0.039	0.007	9.34E-08*
8	*PLEC*	-1231	TSS	cg03958308	145014989	-0.030	0.006	3.40E-06
9	*GLIS3*	71264	BODY	cg14047387	4080919	-0.035	0.007	7.33E-07
9	*MIR2964A*	-1100	TSS	cg00321619	131153847	-0.033	0.007	7.52E-07
10	*CAMK1D*	39248	BODY	cg16894855	12430878	-0.031	0.007	3.59E-06
10	*FRMD4A*	-13	TSS	cg11813497	14372879	0.043	0.009	3.48E-06
10	*MGMT*	-1399	TSS	cg09993459	131264102	-0.030	0.006	1.72E-06
10	*FRG2B*	6347	TSS	cg20219790	135434000	-0.040	0.008	1.09E-06
11	*OR5M10*	364	BODY	cg07850316	56344833	-0.031	0.007	5.43E-06
11	*ARHGAP42*	125042	BODY	cg02008402	100683448	-0.034	0.007	1.62E-06
12	*GRIN2B*	23556	BODY	cg17174980	14109514	-0.032	0.007	5.64E-06
12	*ENSG00000212383*	-44252	TSS	cg11147442	57210954	-0.034	0.007	3.36E-06
12	*CUX2*	259376	BODY	cg00029284	111731203	-0.031	0.006	1.53E-06
12	*ENSG00000213144*	30912	TSS	cg13083057	119663566	-0.035	0.008	3.41E-06
12	*LOC100507055*	438	BODY	cg09444108	133186599	-0.032	0.007	3.35E-06
13	*ENSG00000215881*	27438	BODY	cg20338386	112275793	-0.026	0.005	1.27E-06
14	*TTC7B*	274756	BODY	cg18655025	91008005	-0.042	0.008	6.76E-08*
14	*CCDC88C*	17815	BODY	cg23304605	91866373	0.048	0.010	5.75E-06
14	*MEG3*	1703	BODY	cg08698721	101294147	0.038	0.008	2.92E-06
15	*CYP1A1*	-1266	TSS	cg05549655	75019143	0.065	0.010	2.38E-10*
15	*CYP1A1*	-1319	TSS	cg13570656	75019196	0.067	0.014	1.64E-06
15	*CYP1A1*	-1326	TSS	cg12101586	75019203	0.058	0.013	3.68E-06
15	*CYP1A1*	-1374	TSS	cg22549041	75019251	0.098	0.017	8.88E-09*
15	*CYP1A1*	-1358	TSS	cg11924019	75019283	0.044	0.008	4.78E-08*
15	*CYP1A1*	-1425	TSS	cg18092474	75019302	0.068	0.012	9.95E-09*
15	*LOC338963*	1675	BODY	cg02406469	83381070	-0.037	0.008	3.90E-06
16	*CACNA1H*	-17013	TSS	cg00720047	1186227	-0.031	0.007	3.35E-06
16	*ENSG00000214696*	63479	TSS	cg00253658	54210496	0.077	0.016	9.64E-07
16	*ZFHX3*	90978	BODY	cg05764102	72991344	-0.034	0.007	1.24E-06
16	*VAT1L*	3346	BODY	cg04260557	77825828	-0.035	0.007	1.90E-06

Supplemental Material, Table S1 (cont.). Differential methylation in cord blood DNA in relation to maternal cotinine, top 100 most significant CpGs, sorted by chromosome, position

Chr[a]	Gene	Distance[b]	Location[c]	CpG	Position[d]	Coef[e]	SE[f]	p-value*
16	*ANKRD11*	68054	BODY	cg00169122	89488963	0.028	0.006	5.20E-06
17	*C17orf98*	15	TSS	cg19760250	36997627	-0.094	0.020	2.41E-06
17	*C17orf98*	-30	TSS	cg23290482	36997720	-0.064	0.013	1.90E-06
17	*CCR7*	10231	BODY	cg07479709	38711505	-0.035	0.007	1.78E-06
19	*GMIP*	11941	BODY	cg02293766	19742514	-0.029	0.006	4.96E-06
19	*MIR518B*	-1428	TSS	cg11251554	54204562	-0.032	0.006	2.40E-07
20	*TGM3*	32119	BODY	cg21293537	2308779	-0.034	0.007	1.90E-06
20	*LOC339568*	-1038	TSS	cg07376374	37854477	-0.037	0.008	5.01E-06
20	*ATP9A*	72466	BODY	cg07339236	50312490	-0.053	0.010	1.38E-07
21	*RUNX1*	1920	BODY	cg02869559	36259067	0.079	0.017	2.75E-06
21	*RUNX1*	1746	BODY	cg12477880	36259241	0.163	0.026	7.55E-10*
21	*RUNX1*	1652	BODY	cg00994804	36259383	0.164	0.031	1.31E-07
21	*RUNX1*	1527	BODY	cg06758350	36259460	0.073	0.016	3.52E-06
21	*ETS2*	2152	BODY	cg15892280	40180000	-0.029	0.006	7.74E-07

[a]Chromosome; [b] Distance from CpG to transcription start site of the nearest gene (negative=upstream; positive=downstream); [c] CpG is located in transcription start site (TSS) or in the gene body (BODY); [d] Chromosomal position based on NCBI human reference genome assembly Build 37.3; [e] Regression coefficient from robust linear regression adjusted for maternal age, maternal education, parity, and asthma; [f] Standard error for regression coefficient; * Bonferroni-corrected statistically significant ($p < 1.06x10^{-7}$).

Supplemental Material, Table S2. Differential methylation by cell type (polymorphonuclear cells (PM) compared to mononuclear cells (MN)) in cord blood DNA, and the magnitude of differential methylation by maternal smoking in MoBa (Smokers (S) – Non-smokers (NS)[a]).

CHR	Gene	CpG	Median Methylation (beta)		Percent difference in median methylation by cell type (PM – MN)	Percent difference in median methylation by smoking in MoBa (S – NS)
			PM	MN		
1	*GFI1*	cg10399789	0.844	0.840	0.41	-3.66
1	*GFI1*	cg09662411	0.822	0.845	-2.34	-6.65
1	*GFI1*	cg06338710	0.884	0.882	0.24	-5.81
1	*GFI1*	cg18146737	0.904	0.894	1.05	-12.33
1	*GFI1*	cg12876356	0.826	0.836	-1.02	-11.86
1	*GFI1*	cg18316974	0.928	0.942	-1.35	-7.14
1	*GFI1*	cg09935388	0.806	0.797	0.84	-13.68
1	*GFI1*	cg14179389	0.339	0.324	1.49	-8.61
5	*AHRR*	cg23067299	0.765	0.764	0.08	3.15
5	*AHRR*	cg03991871	0.881	0.862	1.87*	-2.21
5	*AHRR*	cg05575921	0.854	0.851	0.31	-7.52
5	*AHRR*	cg21161138	0.818	0.813	0.46	-2.27
6	*HLA-DPB2*	cg11715943	0.883	0.863	1.97	-1.77
7	*MYO1G*	cg19089201	0.922	0.911	1.04	1.44
7	*MYO1G*	cg22132788	0.956	0.951	0.59	2.82
7	*MYO1G*	cg04180046	0.617	0.586	3.10*	5.30
7	*MYO1G*	cg12803068	0.862	0.844	1.81	8.31
7	*ENSG00000225718*	cg04598670	0.634	0.623	1.06	-3.01
7	*CNTNAP2*	cg25949550	0.213	0.204	0.97	-1.80
8	*EXT1*	cg03346806	0.829	0.818	1.02*	-1.51
14	*TTC7B*	cg18655025	0.888	0.886	0.23	-1.19
15	*CYP1A1*	cg05549655	0.278	0.288	-0.97	3.50
15	*CYP1A1*	cg22549041	0.408	0.424	-1.59	7.23
15	*CYP1A1*	cg11924019	0.566	0.563	0.21	3.23
15	*CYP1A1*	cg18092474	0.627	0.629	-0.20	5.90
21	*RUNX1*	cg12477880	0.098	0.100	-0.18	4.55

* Bonferroni-corrected statistically significant (Bonferroni $p < 0.05$, raw $p < 0.0019$).

[a] Maternal smoking determined by plasma cotinine (nmol/L) measured around gestational week 18. Values above 56.8 nmol/L indicate active smoking during pregnancy.

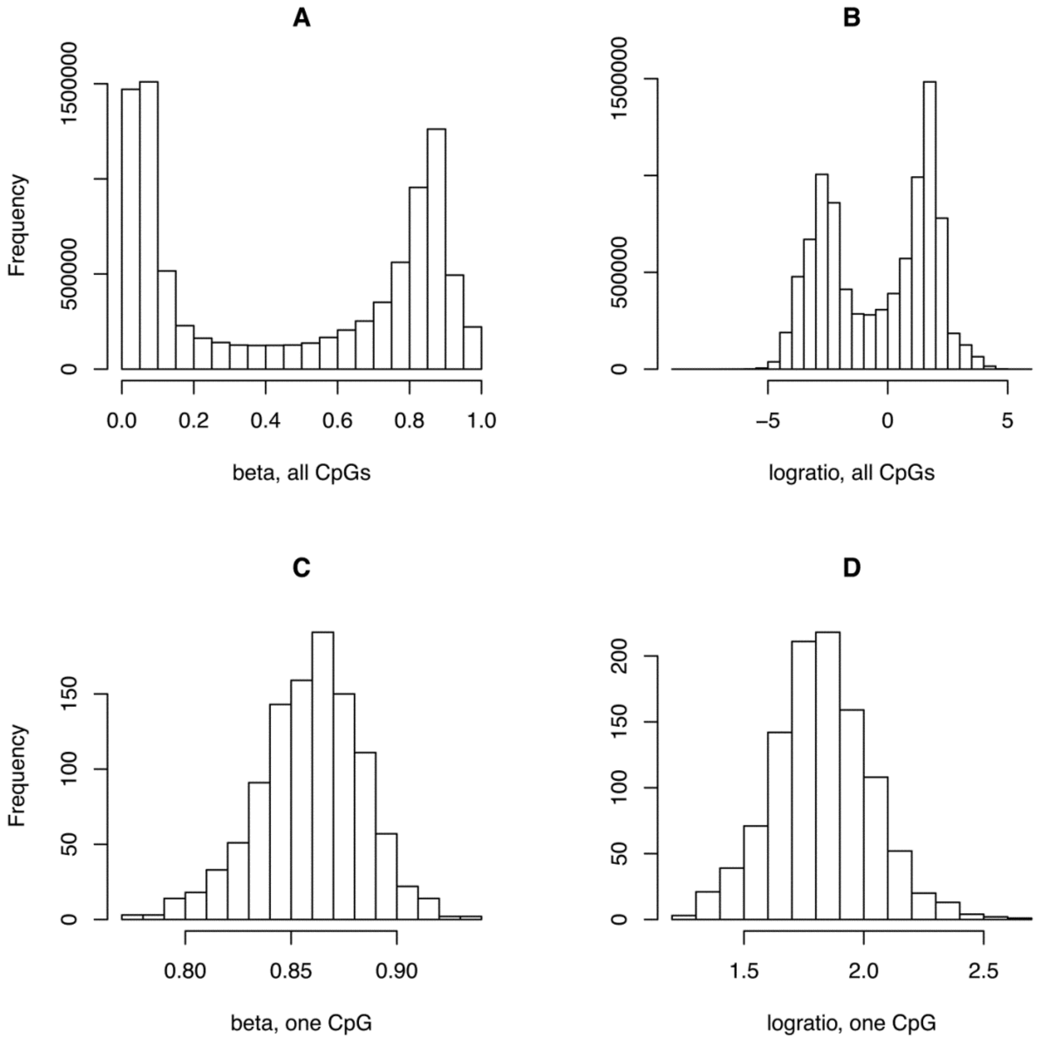

Supplemental Material, Figure S1. Histograms showing the distribution of methylation levels in our data. Bimodal distribution was observed when considering all 473,844 CpG sites whereas approximately normal distribution was observed for most individually plotted CpG sites. (a) Beta across all CpGs analyzed; (b) log(beta/1-beta) across all CpGs analyzed; (c) Beta for one representative CpG (cg11924019); (d) log(beta/1-beta) for the representative CpG.

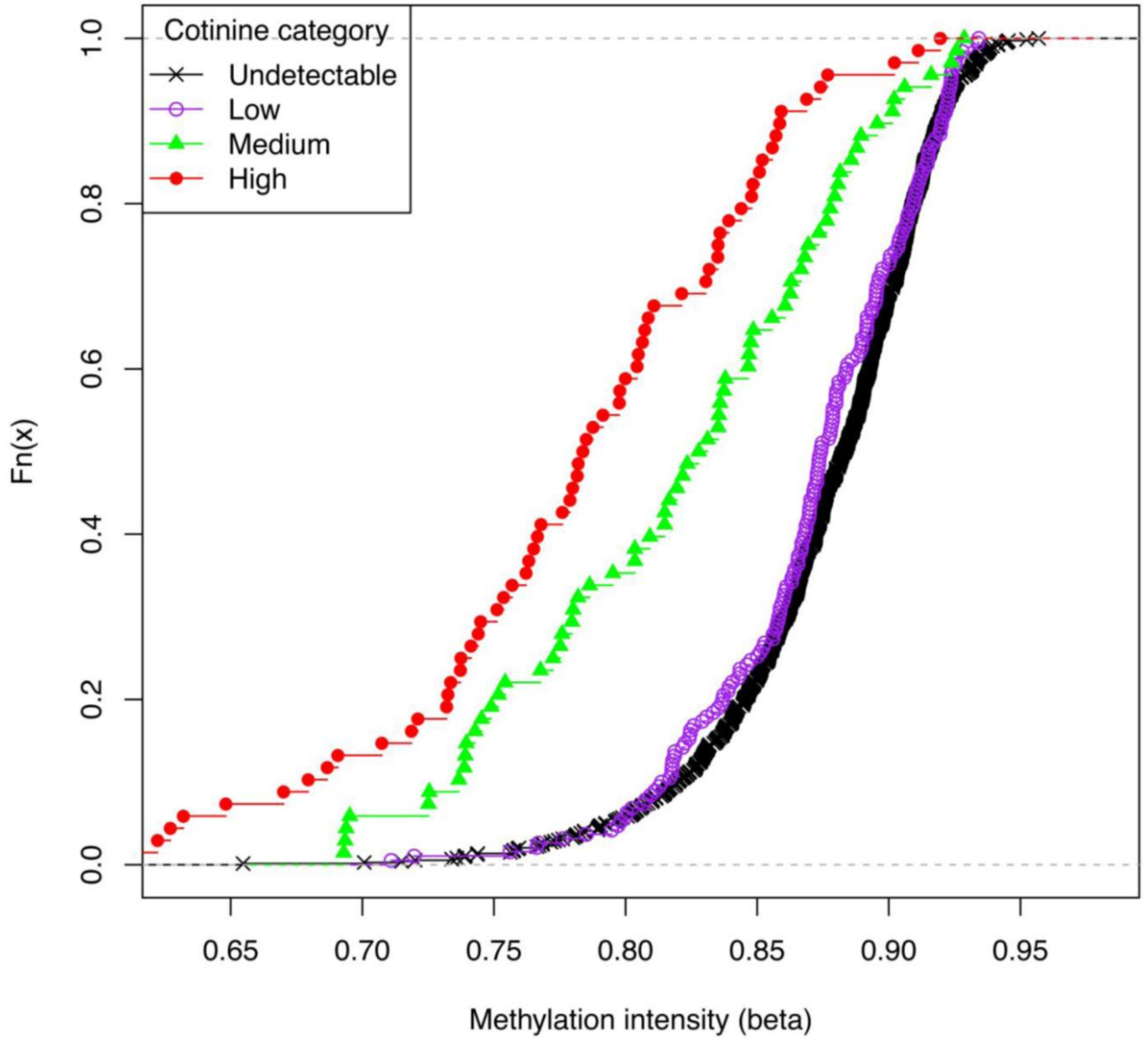

Supplemental Material, Figure S2. Plot of the cumulative distribution function for methylation intensities for the top-ranked CpG site (*AHRR* cg05575921) by the four cotinine categories (undetectable: ≤0 nmol/L; low: >0-56.8 nmol/L; moderate: >56.8 – 388 nmol/L; high: >388 nmol/L) demonstrates a dose-response effect of smoking in the MoBa cohort (Jonkheere-Terpstra trend test $p < 2.2 \times 10^{-16}$). The Jonkheere-Terpstra trend test was calculated using the SAGx package in R, version 2.14.0. Cotinine values above 56.8 nmol/L are consistent with active smoking.

a

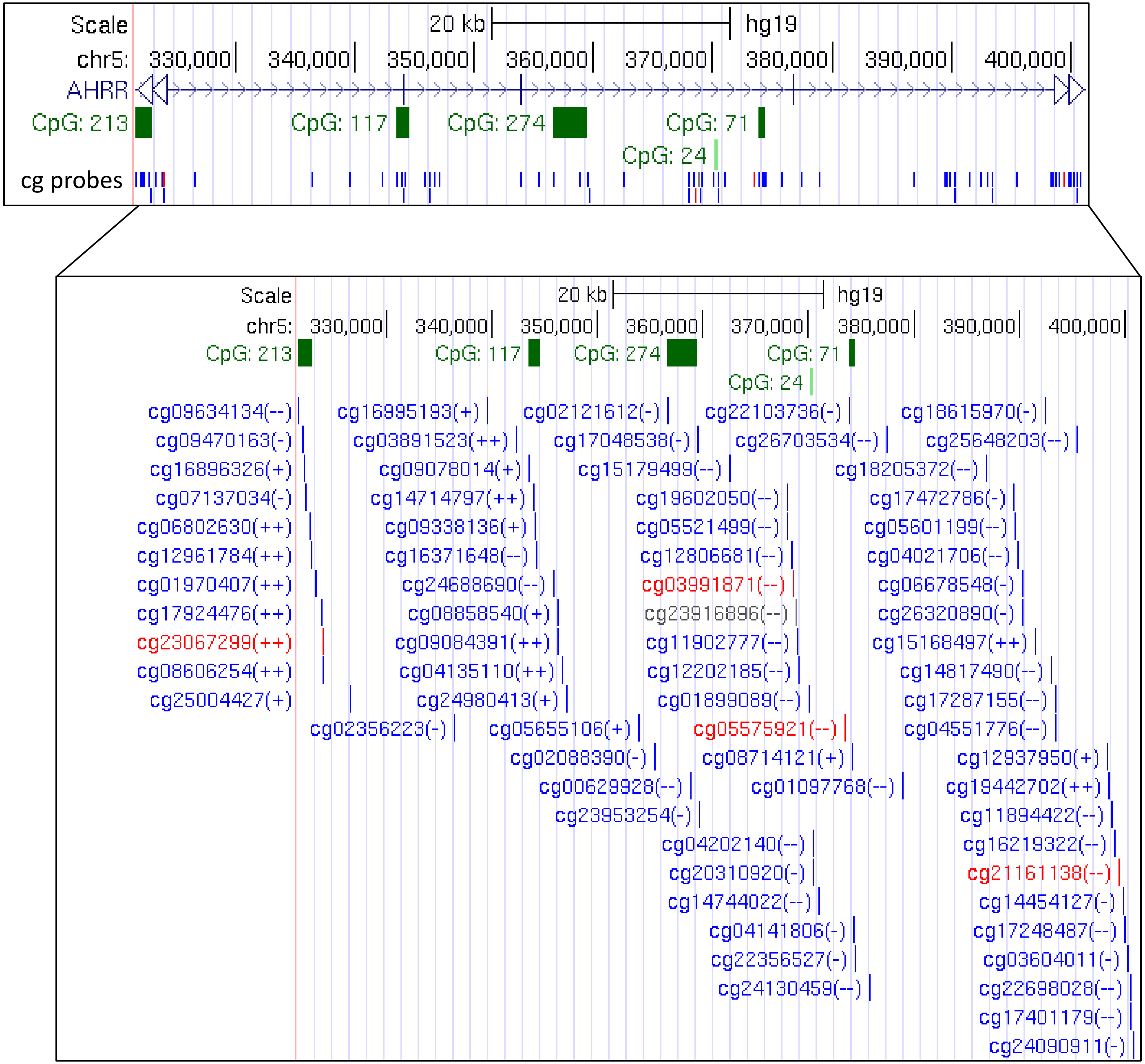

Scale
20 kb
hg19
chr5: 330,000 340,000 350,000 360,000 370,000 380,000 390,000 400,000
AHRR
CpG: 213
CpG: 117
CpG: 274
CpG: 71
CpG: 24
cg probes
cg09634134(--)
cg09470163(-)
cg16896326(+)
cg07137034(-)
cg06802630(++)
cg12961784(++)
cg01970407(++)
cg17924476(++)
cg23067299(++)
cg08606254(++)
cg25004427(+)
cg02356223(-)
cg16995193(+)
cg03891523(++)
cg09078014(+)
cg14714797(++)
cg09338136(+)
cg16371648(--)
cg24688690(--)
cg08858540(+)
cg09084391(++)
cg04135110(++)
cg24980413(+)
cg05655106(+)
cg02088390(-)
cg00629928(--)
cg23953254(-)
cg02121612(-)
cg17048538(-)
cg15179499(--)
cg19602050(--)
cg05521499(--)
cg12806681(--)
cg03991871(--)
cg23916896(--)
cg11902777(--)
cg12202185(--)
cg01899089(--)
cg05575921(--)
cg08714121(+)
cg01097768(--)
cg04202140(--)
cg20310920(-)
cg14744022(--)
cg04141806(-)
cg22356527(-)
cg24130459(--)
cg22103736(-)
cg26703534(--)
cg18205372(--)
cg17472786(-)
cg05601199(--)
cg04021706(--)
cg06678548(-)
cg26320890(-)
cg15168497(++)
cg14817490(--)
cg17287155(--)
cg04551776(--)
cg12937950(+)
cg19442702(++)
cg11894422(--)
cg16219322(--)
cg21161138(--)
cg14454127(-)
cg17248487(--)
cg03604011(-)
cg22698028(--)
cg17401179(--)
cg24090911(-)
cg18615970(-)
cg25648203(--)

b

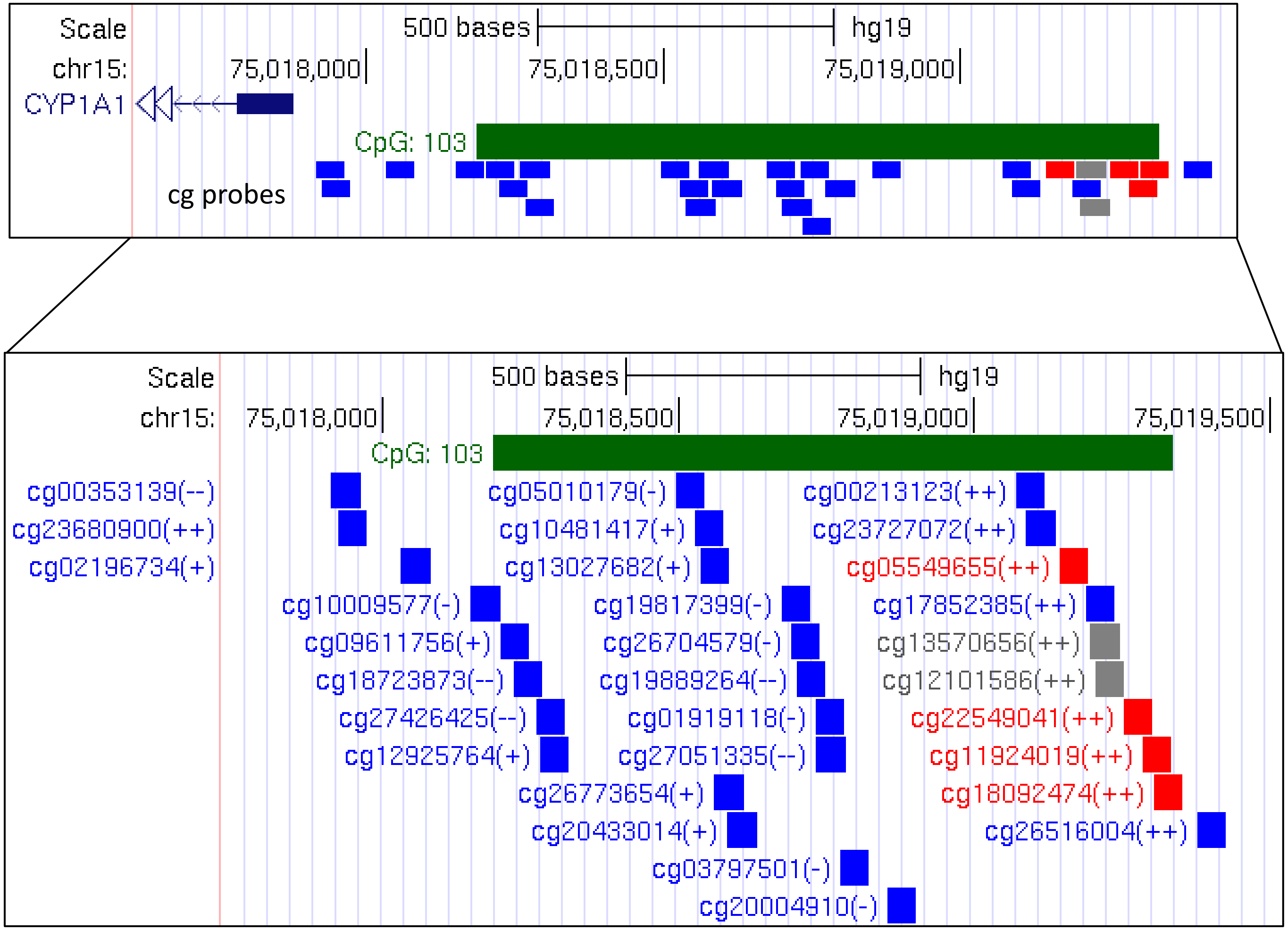

Supplemental Material, Figure S3. (a) CpG sites located in the intron region of the *AHRR* gene. (b) CpG sites located on the shore of a CpG island in a bidirection regulatory region of the *CYP1A* cluster. The statistical significance of the association between cotinine and methylation of each probe is color coded (blue: $p > 1x10^{-5}$; grey: $1x10^{-5} \geq p \geq 1x10^{-7}$; red: $p < 1x10^{-7}$). The magnitude of effect (coefficient from robust linear regression) is indicated as: <-0.01 (--), -0.01 to 0 (-), >0 to 0.01 (+), and >0.01 (++).
